# Real-Time Tissue Classification Using a Novel Optical Needle Probe for Biopsy

**DOI:** 10.1177/00037028241230568

**Published:** 2024-02-19

**Authors:** Lukasz Surazynski, Ville Hassinen, Miika T. Nieminen, Tapio Seppänen, Teemu Myllylä

**Affiliations:** 1 Research Unit of Health Sciences and Technology, Faculty of Medicine, 6370 University of Oulu, Oulu, Finland; 2 Optoelectronics and Measurement Techniques Research Unit, Faculty of Information and Electrical Engineering, 6370 University of Oulu, Oulu, Finland; 3 Center for Machine Vision and Signal Analysis, Faculty of Information and Electrical Engineering, 6370 University of Oulu, Oulu, Finland; 4 Department of Diagnostic Radiology, 60664 Oulu University Hospital, Oulu, Finland

**Keywords:** Diffuse optical spectroscopy, core needle biopsy, classification, smart probe

## Abstract

Core needle biopsy is a part of the histopathological process, which is required for cancerous tissue examination. The most common method to guide the needle inside of the body is ultrasound screening, which in greater part is also the only guidance method. Ultrasound screening requires user experience. Furthermore, patient involuntary movements such as breathing might introduce artifacts and blur the screen. Optically enhanced core needle biopsy probe could potentially aid interventional radiologists during this procedure, providing real-time information on tissue properties close to the needle tip, while it is advancing inside of the body. In this study, we used diffuse optical spectroscopy in a custom-made core needle probe for real-time tissue classification. Our aim was to provide initial characteristics of the smart needle probe in the differentiation of tissues and validate the basic purpose of the probe of informing about breaking into a desired organ. We collected optical spectra from rat blood, fat, heart, kidney, liver, lungs, and muscle tissues. Gathered data were analyzed for feature extraction and evaluation of two machine learning-based classifiers: support vector machine and *k*-nearest neighbors. Their performances on training data were compared using subject-independent *k*-fold cross-validation. The best classifier model was chosen and its feasibility for real-time automated tissue recognition and classification was then evaluated. The final model reached nearly 80% of correct real-time classification of rat organs when using the needle probe during real-time classification.

## Introduction

Core needle biopsy (CNB) is part of histopathological inspection,^1^ in which a hollow organ-specific needle is pushed into the body.^2^ It is followed by a relatively long sample preparation process,^3^ which allows for the differentiation of healthy and diseased tissue, and therefore it is useful in medical diagnosis. Ultrasound screening is a method that is the gold standard to follow the path of the needle during biopsy and visualize its localization inside of the body.^4,5^ Based on the density of tissues and scattering properties of the needle tip, it is possible to track and estimate its path toward the suspected tumor. Unfortunately, access to ultrasound for tissue recognition could be limited, therefore combining optics, also as a label-free method could bring additional benefits to this procedure to improve sampling and save time and resources.^6^

Biopsy by definition is an invasive procedure. Measurements of optical properties of tissues supporting CNB can be realized invasively using a fiber source and a detector embedded in the needle. Fiberoptic needles were already successfully tested in invasive tissue diagnostics. Villiger et al.^7^ proposed a method of deep tissue imaging using birefringence in order to differentiate tumor margins with 24-gauge (570 µm). Amanzadeh et al.^8^ described an overall review of recent developments in shape sensing using fiber optics, where many industrial and medical applications might be found. Optical fibers embedded into steel needles and controlled by a robotic arm were presented in Sareh et al.^9^ in which such a robotic arm could be utilized in surgical instruments with high precision. Zhang et al.^10^ evaluated the construction of a fiberoptic endoscope for three-dimensional high-dose-rate in residual nasopharyngeal carcinoma after conventional external beam radiotherapy. The removal of the deep-seated residual tumor was securely achieved and the refractory tumor in the patient healed uneventfully, without a recurrence during 26 months of follow-up. In frames of construction of single fiberoptic needles, side surface interface design was described in Tsubokawa.^11^ In order to improve signal-to-noise ratio and increase collection efficiency multifiber probes might be built, for example, in Raman spectroscopy.^12^ With closer relation to ultrasounds, an ultrasound transmitter for guidance of minimally invasive fetal surgery was proposed by Xia et al.,^13^ where a fiberoptic-based needle was improving the visibility of the needle in challenging fetal surgeries. Additionally, a tip-sensitive fiber optic Bragg grating was used to measure high-intensity-focused ultrasound fields with high spatial resolution.^14^ Out of the spectroscopic methods, a worthy attempt to localize needle tips under ultrasound imaging could be based on optical fiber hydrophone.^15^

In combination with spectroscopy, various machine learning (ML) methods can be applied, where the aim is to categorize input data or objects into identifiable classes based on features or attributes of the data samples.^16^ ML methods rely heavily on statistics, where models are initially fit or divided into classes using a training data set. Some important applications of pattern recognition (PR) include computer-aided diagnosis (CAD) which at present assists clinicians in making decisions.^17^ CAD has been applied to many types of medical data, such as X-rays, computed tomography images, and electrocardiograms.^18,19^ Since medical data is often difficult to interpret, and mistakes are made even by experienced radiologists, CAD applications could provide valuable supportive information for clinicians’ decision-making. The performance of CAD systems has been very promising,^20,21^ and they may offer huge opportunities in improving healthcare. A biopsy needle that recognizes tissue features and classifies tissue at the tip of the biopsy needle while taking the biopsy sample, could be a potential CAD PR application in the near future.

## Experimental

### Materials and Methods

#### Measurement System

**Figure 1.**
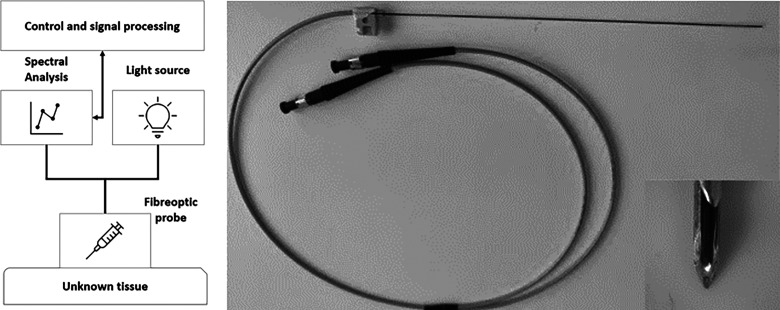
Schematic of the measurement setup (left), optical needle probe with the tip (right).

The spectroscopic measurement system included four main components: light source, a biopsy needle with embedded optical fibers, a spectrometer, and a computer. A schematic diagram of the measurement setup is shown in Figure 1. A halogen–tungsten light source (HL-2000-HP) used in the studies provides 8.8 mW in the range of 360–2400 nm. A standard 18 G core biopsy needle, with an outer diameter of 1.27 mm was modified (Figure 1). Two optical fibers were embedded inside the needle, both having a diameter of 140 µm. The spectrometer (QE Pro, Ocean Insight) covered a wavelength range of 348–1129 nm with a spectral resolution of 0.7 nm. With such a range and resolution, raw spectra contained 1044 wavelengths. The spectrometer was connected to a computer that operates OceanView v.2.0 software (Ocean Insight). The software provided a visual representation of the spectra in real time and allowed continuous repeatable measurements.

### Optical Needle Probe

**Figure 2.**
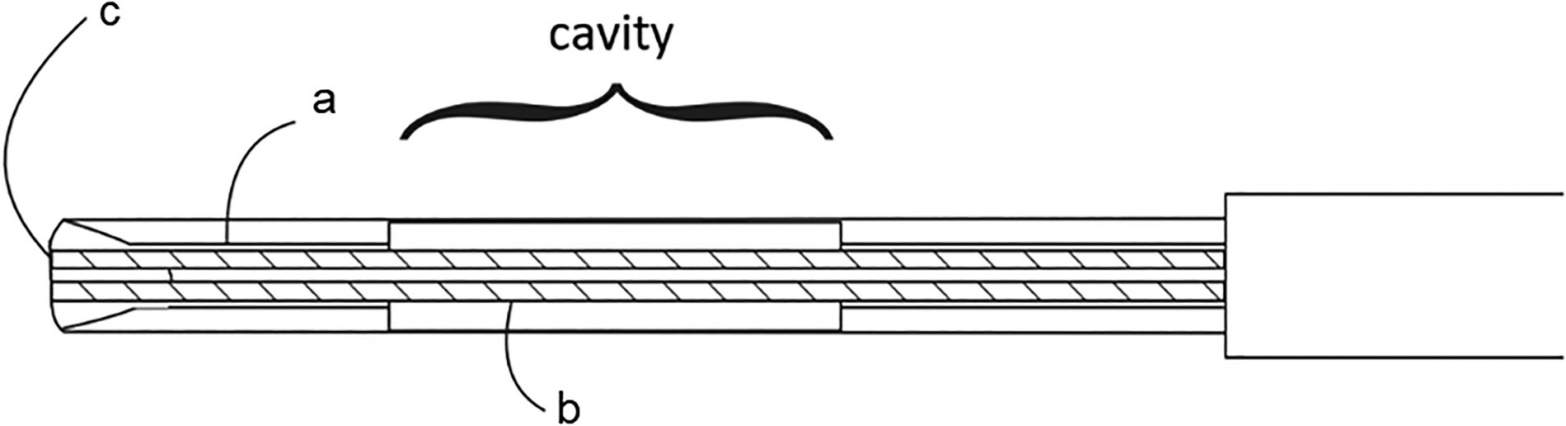
Design of enhanced CNB probe. (a, b) Two optical fibers of 140 µm diameters are embedded on the bottom of the (c) shaft reaching the stylet tip. The distance between the fiber tips is 500 µm.

The spectroscopic probe was built inside a standard 18 G CNB needle (Figure 2). In order to embed two optical fibers longitudinal shaft was laser machined into the stylet. Shaft depth reaches the height of the cavity, which holds the tissue piece after the biopsy procedure. Fiber tips were polished at the same angle as the stylet tip and secured with epoxy glue. In such a configuration screening directly in front of the needle is possible and the sampling process is not disrupted.^22^

### Material and Data

The data-gathering process consisted of standardized steps. At first, rats were anesthetized and euthanized by an Oulu Laboratory Animal Centre veterinarian, and tissues were surgically removed. Extracted tissues were then transported to the measurement laboratory in ice within 3 min. Samples were subsequently placed on a black, wooden measurement board, the biopsy needle was inserted inside of a tissue and the light spectrum in that spot was recorded. All measurements were taken from individual spots of the tissue (Table I). When all possible points were measured, the procedure was repeated within the next rodent.

**Table I.** The number of data samples in each class in the training and model selection and in the validation.

Training and model selection		Validation	
Tissue/class	Amount	Tissue/class	Amount
Ambient light	85	Ambient light	19
Blood	152	Blood	39
Fat	165	Fat	38
Heart	109	Heart	38
Kidney	247	Kidney	38
Liver	201	Liver	38
Lung	121	Lung	38
Muscle	237	Muscle	38
**Total**	**1317**	**Total**	**286**

Table I contains numbers of spectra recorded from six rats that were used for classifier training and model selection. The tissue types (classes) were blood, fat, heart, kidney, muscle, liver, and lungs. Listed tissue samples were considered sufficient to be safely extracted from the animals without losing integrity. In addition to tissue samples, one classifier was expected to recognize laboratory ambient light when the needle is outside the tissue.

The final algorithm was tested in a short and continuous real-time experiment where tissues from additional two rats were extracted and measured ex vivo. In this instance, the measurement setup recorded spectral measurements continuously every 0.5 s. Tissues were measured in the following order: fat, muscle, lung, kidney, liver, heart, and blood due to ruddiness. With this order, the need for needle cleaning between tissues was minimized, thus it was not performed. Changes between tissues were not always perfectly synchronized. To counter this error, samples at class period boundaries were excluded from the analysis. The needle was inserted into tissues through a single spot; when inside, needle depth and angle were slightly adjusted continuously to prevent measuring the same spot for the whole period.

### Data Preprocessing and Partitioning

Optical spectra were imported one by one in a continuous manner. These signals are referred to as raw spectra. In order to obtain relative results between the samples, white standard (WS-1, Ocean Insights) was measured and considered in calculations, together with the noise produced by a spectrometer and the surrounding environment, see Eq. 1:

(1) $$I_{\mathrm{total}}\;[\lambda ]=\frac{I_{\mathrm{raw}}\;-\;I_{\mathrm{noise}}}{I_{\mathrm{standard}}-\;I_{\mathrm{noise}}}\;,\;\mathrm{where}\;I\;=\;\mathrm{light}\;\mathrm{intensity}$$

In order to remove unnecessary high-frequency noise but maintain peaks, spectra were smoothed with a moving average filter with a window size of 10. The intensity of recorded signals varied a lot and as a response min–max normalization was performed to bring the values between 0 and 1 and visually improve changes in spectra. Due to low source emission below 450 nm, low sensitivity of detector above 1000 nm, and no significant differences between classes observed in those bandwidths, spectra were restrained. Limiting the spectra reduces the span of the data, as the signal length was shortened from the previous 1044 down to 732 points.

### Classification Models

For the classification model, data was collected from eight rats. Subject-independent cross-validation (CV) of classifiers was performed in which training data and testing data were kept separate. Six rats were used for algorithm training and model selection, whereas data from two rats was used for real-time classification. There were 1317 spectra collected during the learning phase, where between 85 and 247 spectra were collected from each organ. During real-time classification, a total number of 286 spectra was acquired and 38 out of 39 spectra were from each tissue. A 10-fold stratified CV has been recommended by Berrar for model testing.^23^ However, as the number of spectra for each class was small-scale, a fivefold CV was selected for this study. The number of samples between classes was unbalanced, thus stratification was introduced. After the CV, five different training and validation data sets were obtained. Training data sets contained a total of 1053 out of 1054 spectra in each fold with the number of spectra varying within classes between 68 and 198; in validation data sets, the total number of spectra was 263 out of 264 in each fold with the number of spectra varying withing classes between 17 and 50.

Support vector machine (SVM) and *k*-nearest neighbors (KNN) were two ML classification methods used in this study. There were four feature-specific variations of each classification method. Spectra were either analyzed as full-scale or limited (selected bandwidth, individual points, and slope angles). This resulted in eight classifiers, namely: SVM full-range classifier, KNN full-range classifier, SVM band classifier, KNN band classifier, SVM point classifier, KNN point classifier, SVM slope classifier, and KNN slope classifier. The performances of the eight classifiers were compared with each other in order to choose the final (the most accurate) model. In SVM, the linear kernel function was utilized, as it showed the best accuracy compared to other tested kernels (Gaussian and second-degree polynomial). In KNN, the number of selected neighbors *_k_* = 3, providing the best accuracy with low computation time; when the number of neighbors was increased, performance decreased. Euclidean distance was chosen as the distance metric, as it outperformed Manhattan and Mahalanobis distances.

## Results and Discussion

### Feature Extraction

**Figure 3.**
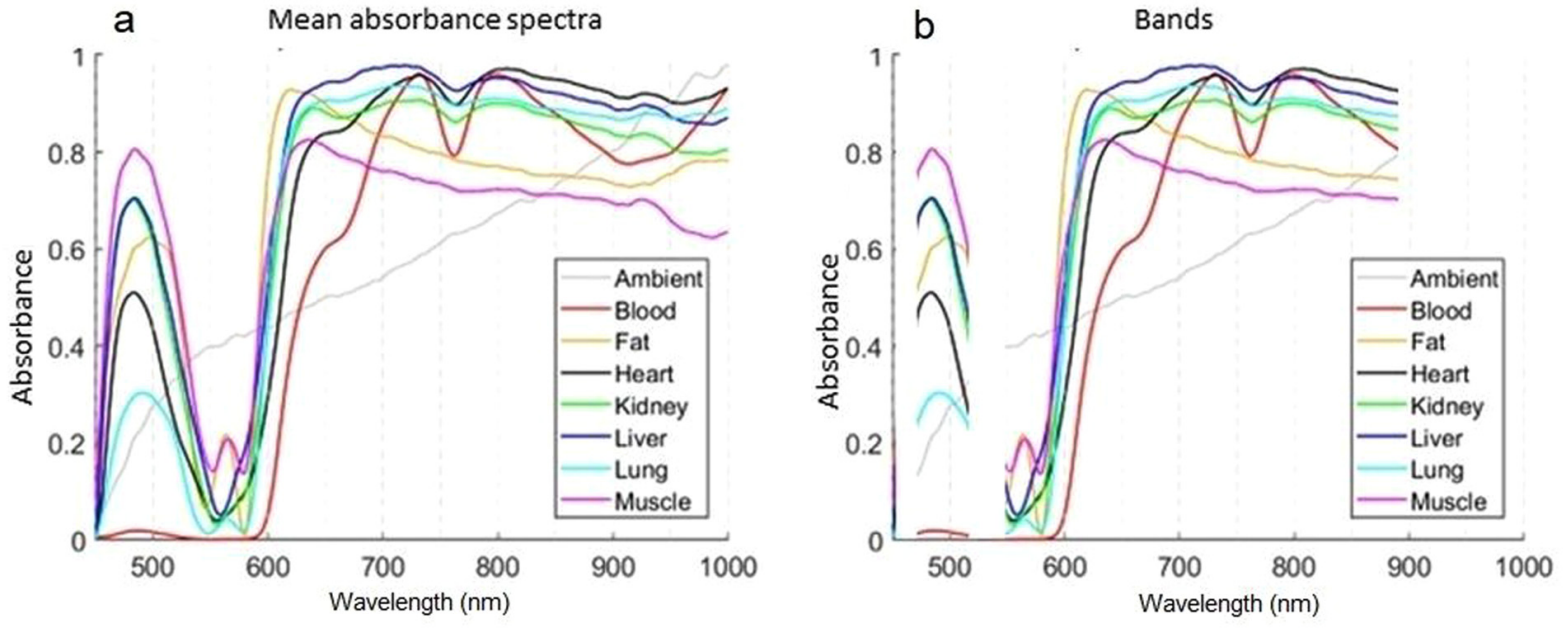
(a) Full-length mean absorbance spectra and (b) selected spectral bands for each class.

Visualization of all recordings for each subsequential class may be found in the Supplemental Material (Figures S1–S8). There were four approaches to extracting features and building classifiers from measured learning spectra. Each subsequential approach reduced the amount of data to be processed. At first, the data set after preprocessing, described in the methodology section, was considered as full length with all the data set's dynamics and possibly redundant parts, see Figure 3a. In the second approach-limited bandwidth, which was the most diverse part of the spectra between different classes was selected. The selected bands, 470–520 nm and 550–890 nm, were chosen based on the visual evaluation. Figure 3b illustrates chosen bands, where the 470–520 nm band contains information on the first wide peak at the beginning of the spectra. The remaining band of 550–890 nm contained an important step rise at around 600 nm with class-specific high dynamics at the end of the bandwidth with a negative peak at 760 nm. After 890 nm, there were no interesting changes in spectral shape observed, except in blood peaks over 900 nm.

The third approach to feature extraction was based on the absorbance values of selected points in the spectra. The idea of the point classifier was to choose the most diverse points and even further limit the amount of data that has to be processed. Table II lists all 25 class-specific feature points. Points were selected in close proximity to rising and falling edges, local maxima, and minima.

**Table II.** Point locations of point classifier. Point columns indicate point numbers. The location column indicates the wavelength location of the point.

Point	Location (nm)
1	485
2	500
3	515
4	545
5	553
6	563
7	570
8	580
9	590
10	600
11	610
12	625
13	640
14	660
15	695
16	730
17	760
18	765
19	800
20	815
21	850
22	900
23	915
24	950
25	990

The fourth approach was based on slope angle-sensitive tangent lines. It was observed that raw absorbance values varied depending on the tissue as they were not homogeneous. Even though the combined values can change inside a class, the spectral shape stays similar. Slopes were calculated between two points. There were a total of 24 slopes/features extracted. Table III shows the starting and ending wavelength locations of each considered slope. With this approach, some information about the signal was lost but the number of features was reduced drastically from 742 to 24.

**Table III.** Start and end locations of each slope in the slope classifier.

Slope	Start (nm)	End (nm)		Slope	Start (nm)	End (nm)		Slope	Start(nm)	End (nm)		Slope	Start (nm)	End (nm)
1	450	475		7	550	565		13	635	650		19	785	800
2	475	485		8	565	580		14	650	670		20	800	880
3	485	500		9	580	590		15	670	700		21	880	900
4	500	515		10	590	600		16	700	730		22	900	940
5	515	530		11	600	620		17	730	760		23	940	970
6	530	550		12	620	635		18	760	785		24	970	1000

### Classifier Testing

Table IV presents classification results for each of the eight classifiers. Each row has the accuracy of classifiers in the corresponding fold and the bottom row contains the average accuracy of classifiers from fivefold. The best accuracy values between folds are highlighted for each metric. The far-right column shows the average accuracy of each fold.

**Table IV.** Classification accuracies of all eight classifier types.

Fold	SVM full range	KNN full range	SVM band	KNN band	SVM slope	KNN slope	SVM point	KNN point	Average
1	0.970	0.829	0.951	0.829	0.962	0.894	0.962	0.837	0.904
2	0.977	0.883	0.951	0.841	0.970	0.947	0.943	0.875	0.923
3	0.962	0.841	0.943	0.830	0.966	0.932	0.936	0.848	0.907
4	0.973	0.814	0.954	0.795	0.973	0.905	0.958	0.821	0.899
5	0.962	0.795	0.905	0.810	0.962	0.897	0.901	0.791	0.878
Average	0.969^a^	0.832	0.941	0.821^b^	0.967	0.915	0.940	0.834	0.902

^a^ Highest average accuracy.

^b^ Lowest average accuracy.

It was evident the SVM full range and SVM slope classifier reached the best-averaged performance, having accuracies of 96.9 and 96.7%, respectively. Additionally, SVM classifiers outperformed KNN classifiers. Thus, in order to find the most optimal model previous SVM classifiers were tested. This phase was also expanded with combinations of classifiers, where the results of two or more classifiers (see Table V) were summed up and averaged. Data set sizes remained the same. However, folds were shuffled, and results are presented in Table V in an analogous manner as previously.

**Table V.** Classification accuracies of combinations of SVM classifiers.

Fold	Full range	Slope classifier	Band classifier	Point classifier	Full range + slope classifier	Full range + slope + band classifier	All four SVM classifiers
1	0.966	0.958	0.951	0.932	0.966	0.970	0.954
2	0.981	0.977	0.958	0.955	0.977	0.977	0.962
3	0.981	0.970	0.951	0.924	0.981	0.977	0.966
4	0.973	0.981	0.932	0.951	0.973	0.973	0.970
5	0.954	0.947	0.943	0.943	0.947	0.947	0.947
Average	0.971^a^	0.967	0.947	0.941^b^	0.969	0.969	0.960

^a^ Highest average accuracy.

^b^ Lowest average accuracy.

It was observed the full-range SVM classifier once again reached the highest average accuracy of 97.1%. The second best-performing classifiers were combinations of full range, slope, and band classifiers. Those were followed by slope classifier and all the above combined. The SVM point classifier was the worst-performing model with an accuracy of 94.1%. Based on these results, a full-range classifier was chosen for the final real-time classification, as it achieved the highest accuracy. The remaining classifiers might have the advantage of training and classification speed due to the lower number of features; however, classification accuracy was prioritized.

### Final Model

The sample rate was fixed to 2 Hz to provide steady recognition during continuous real-time measurements and final model validation. At first, raw spectrum measurement was carried out using the needle probe. Data preprocessing was described in the methodology that took place. A graphical comparison of training and validation data can be found in complementary material.

**Figure 4.**
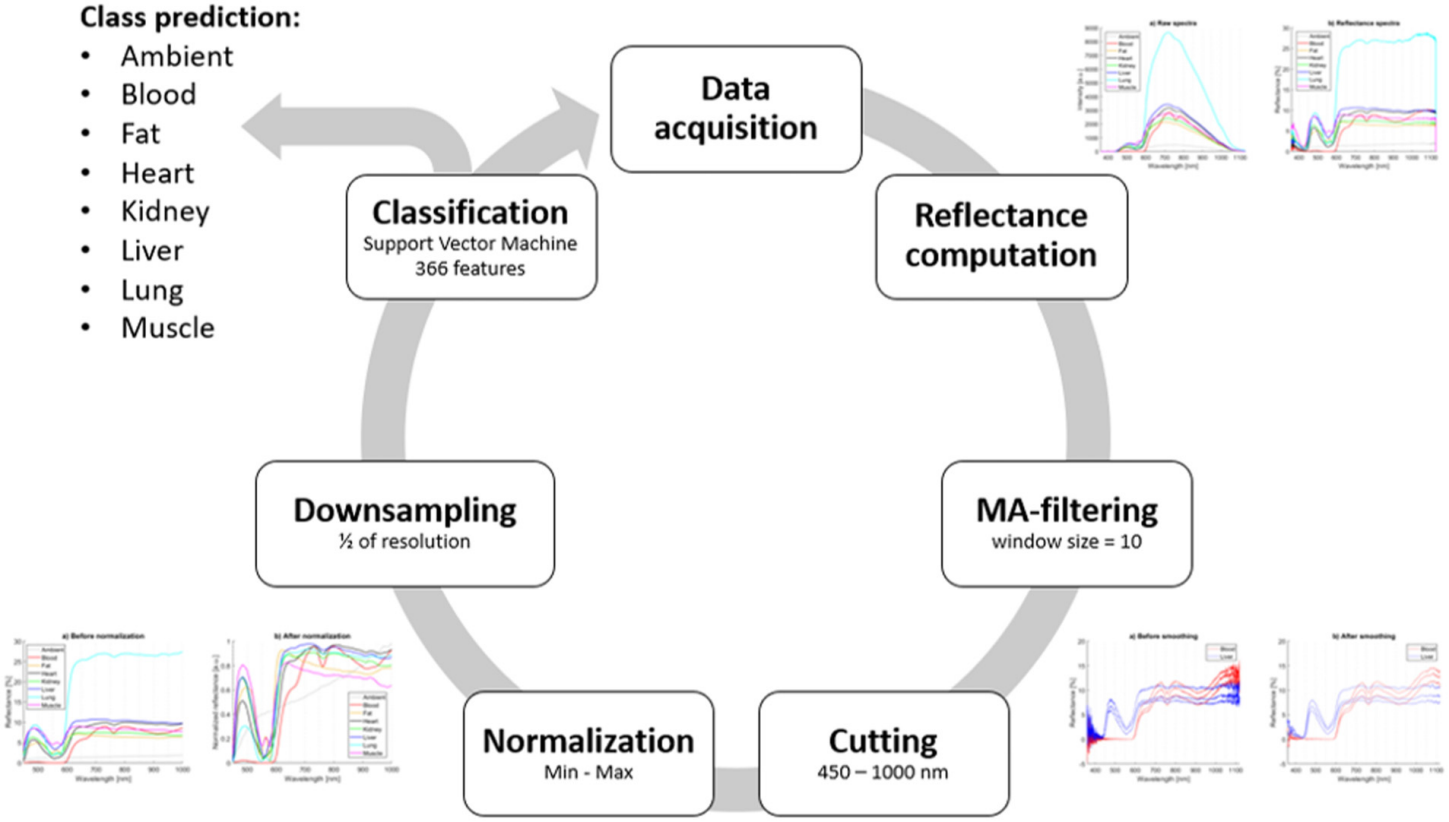
Flow diagram of the final real-time algorithm resulting in class prediction.

Classification by the pre-trained SVM classifier using 366 features and class prediction was the final step. After that, the cycle started from the beginning with the next measured spectrum. On average, 0.26 s were required (ranging between 0.23 and 0.36 s) for final classification using a setup computer. Table VI presents predicted and true classes after real-time classification. Table VII contains tissue-specific performance statistics of real-time testing. Sensitivity, specificity, precision, and F1 score were computed for each tissue separately. The average metrics of all tissues are shown in the bottom row. The best values of each metric are highlighted in green and the worst values are shown in red (Figure 4).

**Table VI.** Predicted and true classes of real-time testing samples.

Class	Prediction								
	Ambient	Blood	Fat	Heart	Kidney	Liver	Lung	Muscle	Total
Blood		35	3				1		39
Fat	1		23					14	38
Heart	2	1	1	26	1		7		38
Kidney			5	3	10		6	14	38
Liver				6		31	1		38
Lung							38		38
Muscle								38	38
Total	2	36	32	35	11	31	53	66	286

Best value of each metric in green.

**Table VII.** Tissue-specific performance statistics of real-time testing.

Class	Sensitivity	Specificity	Precision	F1 score
Blood	0.897	0.996	0.972	0.933
Fat	0.605	0.964	0.719	0.657
Heart	0.684	0.964	0.743	0.712
Kidney	0.263	0.996	0.909	0.408
Liver	0.816	1.000	1.000	0.899
Lung	1.000	0.940	0.717	0.835
Muscle	1.000	0.887	0.576	0.731
Average	0.783	0.967	0.812	0.763

Green values were the best values of each metric and red values were the lowest achieved values.

An enhanced probe provided for biopsy favorable spatial accuracy. Due to the short distance between optical fibers, penetration of the light is limited. Thus, only tissue very close to the needle tip is measured. We approximate it to about 1–2 mm depth. Theoretically, an enhanced needle should be able to spot even thin tissue layers such as fascia. Moreover, a simulation of the biopsy procedure, where the needle is going across tissue layers inside of the organism (e.g., skin, fat, muscle, fat, and specific organs) would be a natural step for needle validation during biopsy. In some instances, such classification could reach better results. More studies are required to support this statement. In this publication, we focused more on the general recognition of tissues/organs.

Results of real-time classification have shown that the overall accuracy during real-time testing reached 76.9% which is not nearly as good as the accuracy achieved during classification model selection (97.1%). Between all classes, average sensitivity, specificity, precision, and F1 score, were 78.3%, 96.7%, 81.2%, and 76.3%, respectively. There was a lot of variation between the class performances. Performance values varied between 26.3 and 100% in sensitivity, 88.7 and 100% in specificity, 57.6 and 100% in precision, and 40.8 and 93.3% in F1 score.

Blood was one of the best-performing classes with good results in all metrics and the highest F1 score of 93.3%. It had only one false positive (FP) and four false negatives (FNs). Blood spectral shape was unique compared to other classes; thus, good performance is not surprising.

Fat results indicate low performance with all metrics. It was misclassified as muscle 14 times, once as ambient, and nine times some other class was classified as fat (Table VI). It was evident the training spectra for fat varied greatly without very distinctive differences. Surprisingly, fat spectra were very similar to muscle, which explains why fat was classified as muscle so many times.

Classification of the heart was performed inadequately, having sensitivity and F1 score of 68.4 and 71.2%, respectively. It was misclassified 12 times, from which it was classified seven times as lungs, once as kidney, and once as liver. It was observed that heart training data varied a lot, which is probably due to the inhomogeneous nature of the organ, and the presence of cavities. Most of the testing spectra had shapes similar to training data. Nonetheless, many spectra with unknown shapes and some spectra similar to lungs were recorded. During final measurements, slipperiness and overall size of the rat heart caused some issues in inserting the needle inside the tissue correctly which could have led to more erroneous data compared to other classes.

From all classes, kidney clearly reached the lowest sensitivity and F1 score of 26.3 and 40.8%, respectively. Kidney samples were classified correctly only 10 times and misclassified 28 times, of which 14 were classified as muscle. Kidney training data encountered challenges as its spectra have similarities with muscle, heart, and liver, explaining why kidney was misclassified so often. The presence of blood was observed in all listed organs and thus, it might have been challenging for the classification algorithm.

Liver performed well, having zero FPs and being the only class with 100% specificity and precision. However, it was misclassified as heart six times and once as lungs.

Lung was the best-performing tissue classifier with 100% sensitivity and zero FNs. It had a low precision score, as four other classes were classified as lung 15 times in total from which seven were heart spectra.

The muscle had 100% sensitivity and zero FNs, which is an excellent result. However, fat and kidney were misclassified as muscle 14 times, respectively. This led to the poorest specificity and precision of all classes. Muscle size and structure varied, meaning that the tissue is not very homogeneous which might cause data variance. Testing data has some variance, but the general shape of the spectra remained the same.

Traditional ML methods were used instead of deep learning (DL) methods, because the amount of available data was relatively small. With big data, DL-based algorithms usually have higher performance than traditional ML algorithms.^24^ With less complex data, DL performance might not be any better than with traditional ML or be even worse. ML methods were considered very useful in reaching our objective, as tissue recognition is a statistical PR problem. Different tissues attenuate light in different ways, and this could be seen as patterns that are characteristic of different tissues.

## Conclusion

Support vector machine (SVM)-based algorithms performed far better than KNN-based algorithms in the classification of rat tissues ex vivo utilizing diffuse optical spectroscopy and our optical probe. The final real-time classification algorithm reached nearly 80% sensitivity, whereas during the learning phase obtained sensitivity was above 94%. Such differences might have happened due to challenging regimes during real-time evaluation. Nonetheless, further improvements are necessary. Especially enlarging the training database could positively impact final results and bring them closer to 94–97%.

## Supplemental Material

sj-docx-1-asp-10.1177_00037028241230568 - Supplemental material for Real-Time Tissue Classification Using a Novel Optical Needle Probe for Biopsy Supplemental material, sj-docx-1-asp-10.1177_00037028241230568 for Real-Time Tissue Classification Using a Novel Optical Needle Probe for Biopsy by Lukasz Surazynski, Ville Hassinen, Miika T. Nieminen, Tapio Seppänen and Teemu Myllylä in Applied Spectroscopy
