# Supplementary material for: Real-Time Tissue Classification Using a Novel Optical Needle Probe for Biopsy
Source: Appl Spectrosc. 2024 Feb 19;78(5):477–85. doi: 10.1177/00037028241230568 (PMC11070118; doi:10.1177/00037028241230568)
Supplement: sj-docx-1-asp-10.1177_00037028241230568 - Supplemental material for Real-Time Tissue Classification Using a Novel Optical Needle Probe for Biopsy [file sj-docx-1-asp-10.1177_00037028241230568.docx]

**Supplemental Material**

**Real-Time Tissue Classification Using a Novel Optical Needle Probe for Biopsy**

Lukasz Surazynski^1,2^,* Ville Hassinen^2^, Miika T. Nieminen^1,4^, Tapio Seppänen^3^, Teemu Myllylä^2^

^1^University of Oulu, Research Unit of Health Sciences and Technology Faculty of Medicine, Aapistie 5 A, Oulu 90220, Finland

^2^University of Oulu, Unit of Optoelectronics and Measurement Technology, Faculty of Information and Electrical Engineering, Erkki Koiso-Kanttilankatu 3, Oulu 90570, Finland

^3^University of Oulu, Center for Machine Vision and Signal Analysis, Faculty of Information and Electrical Engineering, Erkki Koiso-Kanttilankatu 3, Oulu 90570, Finland

^4^Oulu University Hospital, Department of Diagnostic Radiology, Kajaanintie 50, Oulu 90220, Finland

***Corresponding author email:** Lukasz.Surazynski@oulu.fi


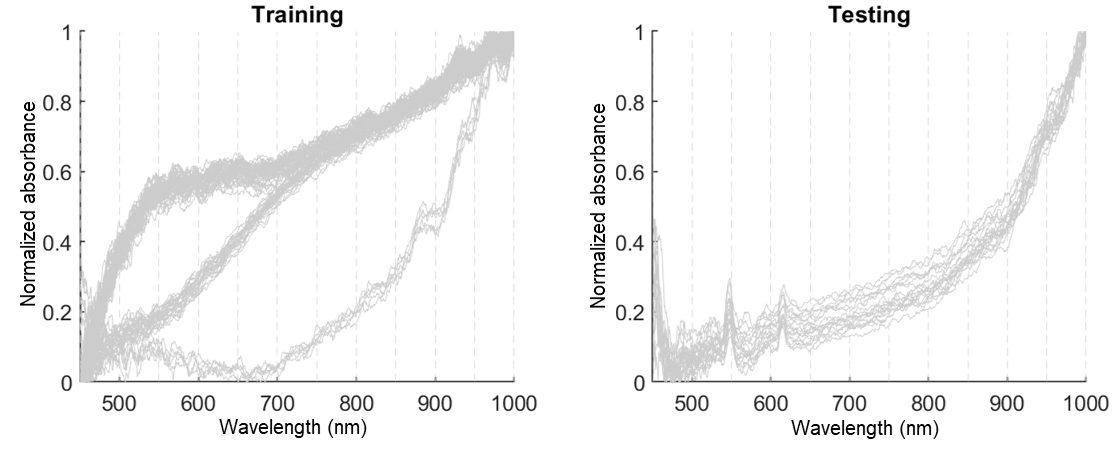


**Figure S1.** Ambient reflectance spectra comparison between training and real-time testing datasets.


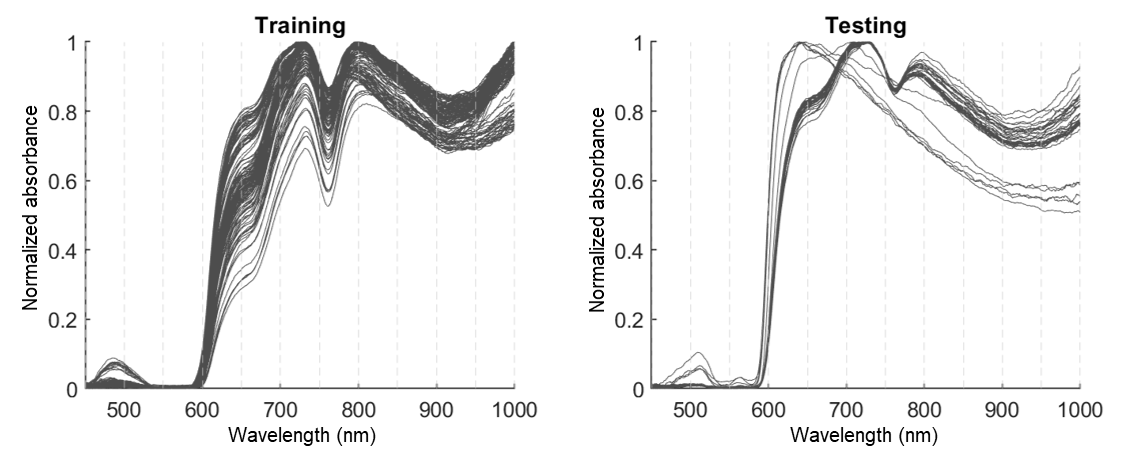


**Figure S2.** Blood reflectance spectra comparison between training and real-time testing datasets.


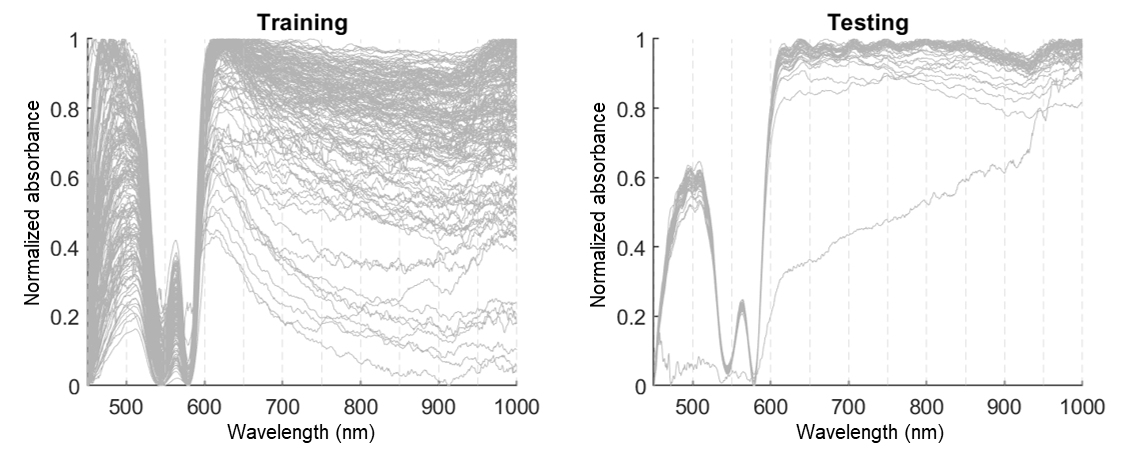


**Figure S3.** Fat reflectance spectra comparison between training and real-time testing datasets.


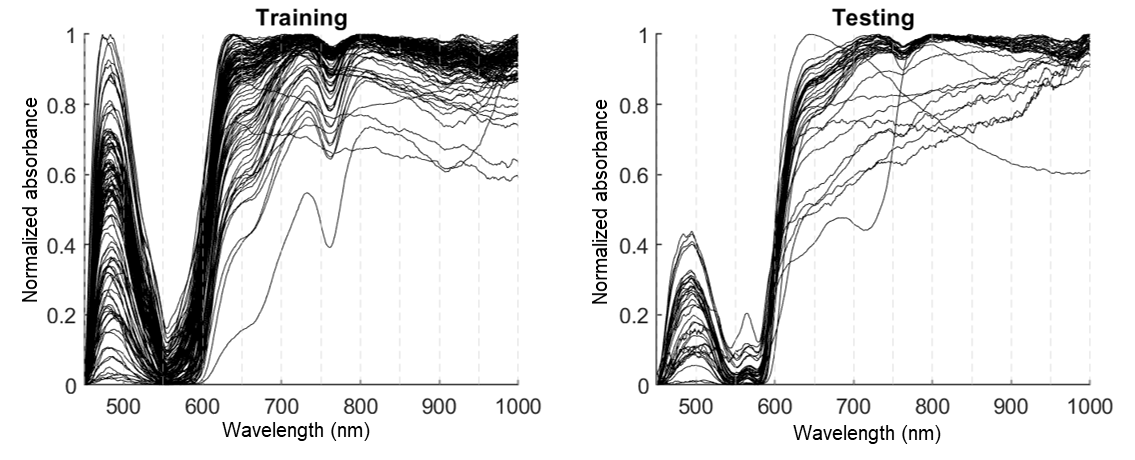


**Figure S4.** Heart reflectance spectra comparison between training and real-time testing datasets.


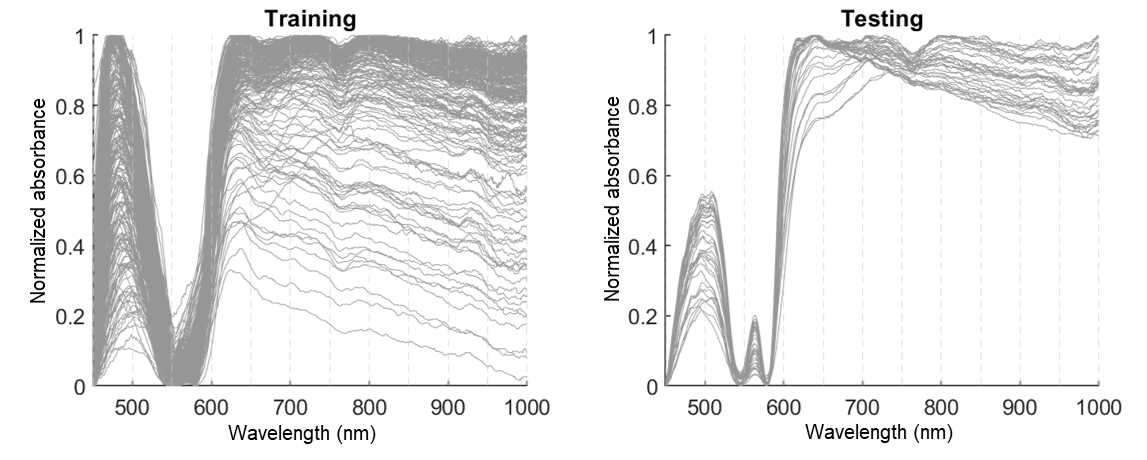


**Figure S5.** Kidney reflectance spectra comparison between training and real-time testing datasets.


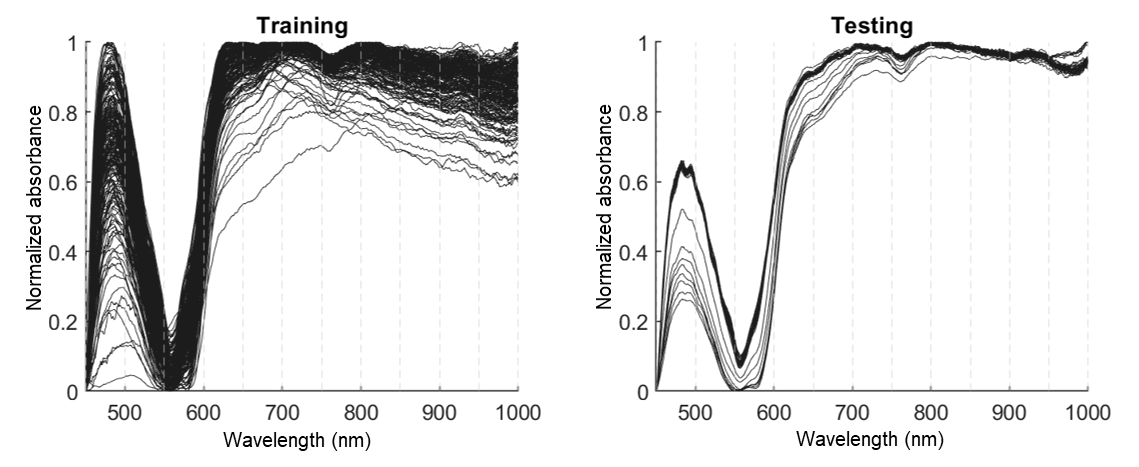


**Figure S6.** Liver reflectance spectra comparison between training and real-time testing datasets.


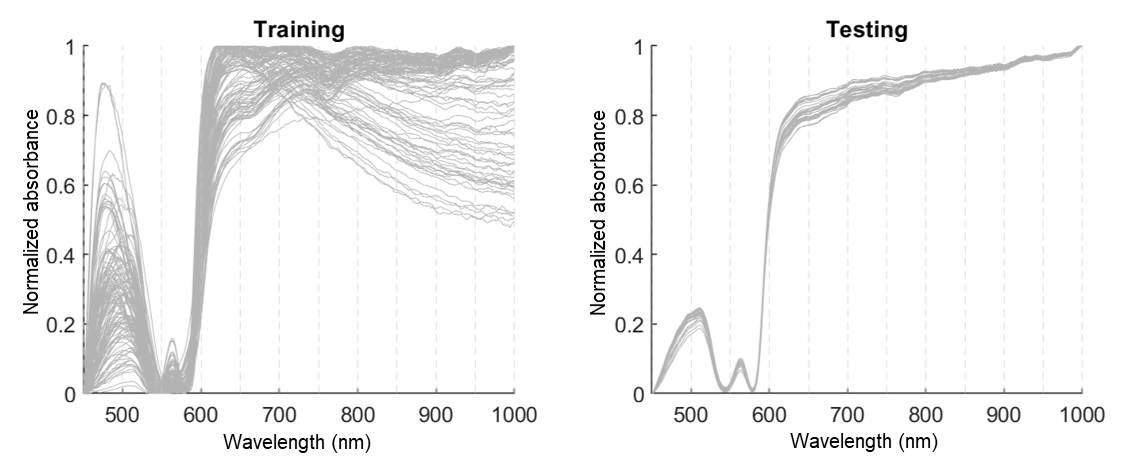


**Figure S7.** Lung reflectance spectra comparison between training and real-time testing datasets.


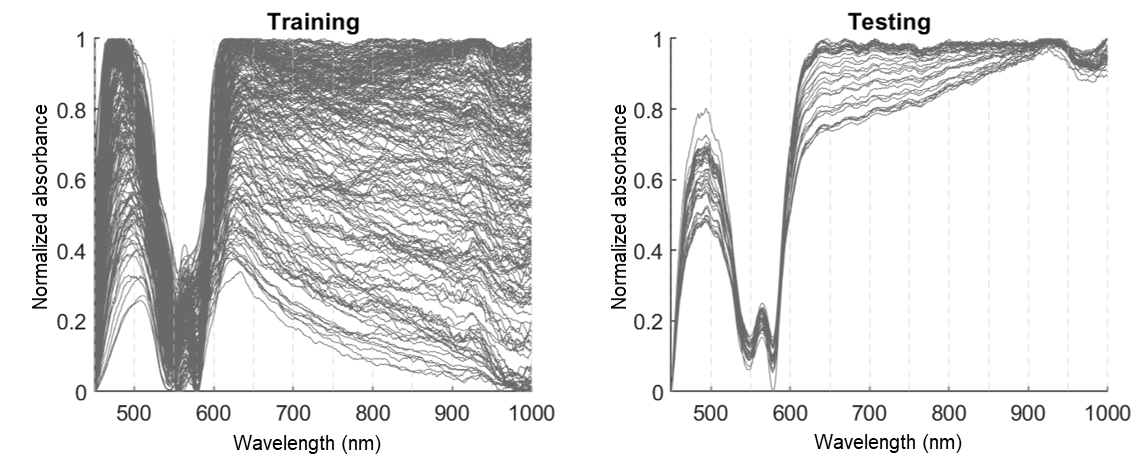


**Figure S8.** Muscle reflectance spectra comparison between training and real-time testing datasets.
